# Acute and chronic HBV infection in central Argentina: High frequency of sub-genotype F1b, low detection of clinically relevant mutations and first evidence of HDV

**DOI:** 10.3389/fmed.2022.1057194

**Published:** 2023-01-09

**Authors:** Gonzalo M. Castro, María J. Sosa, Paola E. Sicilia, María I. Riberi, Claudia Moreno, Rodolfo Cattaneo, José D. Debes, María G. Barbás, Analía E. Cudolá, María B. Pisano, Viviana E. Ré

**Affiliations:** ^1^Departamento Laboratorio Central, Ministerio de Salud de la Provincia de Córdoba, Córdoba, Argentina; ^2^Laboratorio de Virología, Servicio de Microbiología, Clínica Universitaria Reina Fabiola, Universidad Católica de Córdoba, Córdoba, Argentina; ^3^Servicio de Gastroenterología, Hospital Rawson, Ministerio de Salud de la Provincia de Córdoba, Córdoba, Argentina; ^4^Department of Medicine, University of Minnesota, Minneapolis, MN, United States; ^5^Laboratorio de Hepatitis Virales, Instituto de Virología “Dr. J. M. Vanella” (InViV)–CONICET, Facultad de Ciencias Médicas, Universidad Nacional de Córdoba (UNC), Córdoba, Argentina

**Keywords:** hepatitis B virus, HBV, antiviral resistance, mutant, genotypes, Argentina

## Abstract

**Introduction:**

Genomic analysis of hepatitis B virus (HBV) identifies phylogenetic variants, which may lead to distinct biological and clinical behaviors. The satellite hepatitis D virus (HDV) may also influence clinical outcomes in patients with hepatitis B. The aim of this study was to investigate HBV genetic variants, including clinically relevant mutations, and HDV infection in acute and chronic hepatitis B patients in central Argentina.

**Methods:**

A total of 217 adult HBV infected patients [acute (AHB): *n* = 79; chronic (CHB): *n* = 138] were studied; 67 were HBV/human immunodeficiency virus (HIV) coinfected. Clinical and demographic data were obtained from medical records. Serological markers were determined. Molecular detection of HBV and HDV was carried out by RT-Nested PCR, followed by sequencing and phylogenetic analysis.

**Results:**

Overall, genotype (gt) F [sub-genotype (sgt) F1b] was the most frequently found. In AHB patients, the gts/sgts found were: F1b (74.7%) > A2 (13.9%) > F4 (7.6%) > C (2.5%) > A1 (1.3%). Among CHB patients: F1b (39.1%) > A2 (23.9%) > F4 (18.2%) > D (9.4%) > C and F6 (3.6% each) > A1, A3 and B2 (0.7% each). The distribution of sgt A2 and gt D was significantly different between HBV mono and HBV/HIV coinfected patients [A2: 15.9% vs. 35.7% (*p* < 0.05), respectively and D: 14.6% vs. 1.8% (*p* < 0.05), respectively]. Mutation frequency in basal core promoter/pre-Core (BCP/pC) region was 35.5% (77/217) [AHB: 20.3% (16/79), CHB: 44.2% (61/138)]. In the open reading frame (ORF) S, mutations associated with vaccine escape and diagnostic failure were detected in 7.8% of the sequences (17/217) [AHB: 3.8% (3/79), CHB: 10.1% (14/138)]. ORF-P amino acid substitutions associated with antiviral resistance were detected in 3.2% of the samples (7/217) [AHB: 1.3% (1/79), CHB 4.3%, (6/138)]. The anti-HDV seropositivity was 5.2% (4/77); one sample could be sequenced, belonging to gt HDV-1 associated with sgt HBV-D3.

**Discussion:**

We detected an increase in the circulation of genotype F in Central Argentina, particularly among AHB patients, suggesting transmission advantages over the other genotypes. A low rate of mutations was detected, especially those with antiviral resistance implications, which is an encouraging result. The evidence of HDV circulation in our region, reported for the first time, alerts the health system for its search and diagnosis.

## 1. Introduction

Hepatitis B virus (HBV) infection is currently one of the main public health problems worldwide. This is a human pathogen that leads to both self-limited and chronic infections. Despite the availability of a safe and effective vaccine, the World Health Organization (WHO) estimates that 296 million people were living with chronic hepatitis B infection in 2019, with 1.5 million new infections each year (1).

Currently, 10 HBV genotypes (gt) (A to J), -which present >8% genetic divergence- and various sub-genotypes (sgt) -presenting >4% genetic divergence- have been described (2). Determining the viral gt, sgt and isolate is useful to understand the evolution and the epidemiology of the virus. Several clinical and epidemiological observations suggest that genetic differences in viral gts may underline differences in biological and clinical parameters (3, 4).

Changes in the molecular genotype profile over time can be due to multiple factors, in addition to those inherent to the evolutionary advantages between variants. Population movements that favor the introduction of new variants, which in turn can generate new recombinant strains, changes in cultural/social patterns that favor or hinder certain modes of transmission, global human migrations that introduce genotypes differing from those found in the original inhabitants, the most efficient antiviral treatments for some genotypes than for others, might have been involved (5, 6).

Specific mutations have been described in diverse parts of the HBV genome. Nucleotide changes in the Basal Core Promoter (BCP) and preCore (pC) regions, which are associated with the regulation and expression of hepatitis B “e” antigen (HBeAg), have been associated with more severe clinical courses (7–9). Mutations that cause a conformational change in the “a” determinant of the hepatitis B surface antigen (HBsAg) may affect protein antigenicity, essential for inducing neutralizing antibodies, and be responsible for preventing vaccine- or anti-HBV immunoglobulin-induced immunity and providing false-negative results in serological tests (10–12).

Hepatitis delta virus (HDV) is a satellite virus of HBV. Globally, nearly 5% of people who have chronic hepatitis B are HDV positive (13). Co-infection or superinfection with HDV is considered the most severe form of chronic viral hepatitis due to more rapid progression toward liver-related disease and hepatocellular carcinoma (14). Eight HDV genotypes have been identified all over the world, each of which might have a different clinical outcome (14). While HDV genotypes 1 and 3 have been associated with lower remission rates and a more adverse clinical outcome (15). There is no specific interaction between HBV and HDV genotypes, and the combination of genotypes seems to simply reflects the most common genotypes circulating in a given region (16, 17).

In Argentina, the distribution of HBV genotypes has been changing over time, and has reflected the population movements that have occurred in our territory. Genotypes F (the major genotype found), A, B, C, and D have been described, with frequencies that vary according to the geographic region and the population studied (18–27).

Few investigations have been carried out in our region regarding biological and clinical implications of the circulating genotypes and sub-genotypes. More recently, Di Lello et al. (25), found vaccine escape mutations, diagnostic failure mutations, and antiviral resistance mutations in 7.5, 10.7, and 5.1% of cases, respectively. In relation to HDV, there are very few reports. Two previous studies show HDV-1 detection in the Amerindian population of northeastern of Argentina (28) and in blood donors from the Buenos Aires province (22).

In order to deepen the studies of HBV in our country, the aim of the present study was to investigate the infecting genotype, sub-genotype and clinically relevant mutations in acute and chronic HBV infections in the central region of Argentina. Additionally, we investigated HDV infections.

## 2. Materials and methods

### 2.1. Study population

A cross-sectional, observational, and retrospective study was conducted on 217 HBV adult infected patients determined by the presence of HBsAg, who had access to public health centers of Cordoba (the second most populated inland province of Argentina), between 2010 and 2017.

Serum samples were classified as acute hepatitis B (AHB, *n* = 79) or chronic hepatitis B (CHB, *n* = 138). Diagnostic criteria for AHB were as follows: acute onset of symptoms without a history of chronic HBV infection, levels of serum alanine aminotransferase (ALT) >10-fold the upper reference limit, positivity for IgM antibody to the hepatitis B core antigen (anti-HBc), a rapid drop of HBsAg titer, serum HBV-DNA elimination and HBeAg seroconversion at convalescent phase. The diagnosis was confirmed by HBsAg clearance within 6 months after the initial onset. CHB met the following criteria: HBsAg positivity for more than 6 months.

HIV-infected patients were included. Patients were divided into two groups: patients with acute HBV infection (AHB: HBV + / HIV-, *n* = 68 and HBV + /HIV + , *n* = 11) and chronic patients (CHB: HBV + / HIV-, *n* = 82 and HBV + /HIV + , *n* = 56).

Demographic data (age, gender, and HBV viral load) were obtained from medical records. Antiretroviral therapy data were obtained from 114 chronic patients. Fibrosis score (Metavir F0-4) and inflammatory activity in liver tissue data were available from 30 chronic patients.

### 2.2. Serological markers of HBV infection and HBV viral load determination

The following serological markers were evaluated by chemiluminescent microparticle immunoassay (CMIA) on the ARCHITECT system (Abbott Diagnostics, USA): HBeAg; HBsAg; IgM anti-HBc; anti-HBe. HBV DNA levels were determined using the COBAS^®^ TaqMan^®^ HBV Test (Roche Diagnostics, Germany), targeting the highly conserved pre-Core/Core region of the HBV genome (limit of detection: 29 UI/mL). Samples with HBV viral load greater than the maximum quantification limit were diluted and reprocessed until the exact viral load value was obtained.

### 2.3. Anti-HDV antibody detection

Total anti-HDV antibodies were assessed in 77 HBsAg (+) samples, 3 from AHB patients and 74 from CHB patients, using the enzyme immunoassay (EIA) ETI-AB-DELTAK-2 (DiaSorin, Italy).

### 2.4. Molecular detection and sequencing of HBV and HDV

Nucleic acid extraction was performed using the High Pure Viral Nucleic Acid Kit (Roche Diagnostics, Germany), strictly following the manufacturer’s instructions. For HBV, the S gene and the BCP-pC gene regions were amplified (585 bp and 742 bp, respectively) using the protocol described by Pisano et al. (20) (Supplementary Table 1).

For HDV molecular detection, a reverse transcription using the ImProm-II*™* Reverse Transcription System (Promega, USA), and random hexamer primers, followed by a Nested-PCR for amplification of a 353 bp genomic fragment of the HDAg was carried out (22) (Supplementary Table 1).

In all cases, PCR products were purified using the PureLink*™* Quick Gel Extraction Kit (Invitrogen, USA). Direct nucleotide sequencing reaction in both directions was carried out using a 3500xL Genetic Analyzer (Applied Biosystems, USA), using a locally standardized and validated protocol with the same primers used in amplification stages.

### 2.5. Phylogenetic analysis

Phylogenetic analyses were performed using the maximum likelihood method with the software MEGA (v6.0) (29), under the appropriate nucleotide substitution model selected by jModeltest (30), according to the Akaike Information Criterion. The robustness of the reconstructed phylogenies was evaluated by bootstrap analysis (1000 replicates). For HBV, the analysis was performed by combining the sequences obtained from the S and BCP-pC genes, while for HDV, we used the sequence from the HDAg fragment.

### 2.6. Analyses of mutations in ORF-S, ORF-P, and BCP-pC genomic regions

The HBV nucleotide and amino acid sequences were aligned and compared with the prototype strains of each sub-genotype using the MEGA (v6.0) (29) program, the Mutation Reporter Tool (31), and Geno2pheno HBV tools from the Max Planck Institute.^1^ Amino acid sequences corresponding to the ORF-S and ORF-P genes (using sequences obtained from the S gene), as well as the ORF-pC/C (using sequences obtained from BCP-pC) were analyzed.

In order to search for the most significant HBV surface mutants, aa 99–169 within the HBsAg gene were examined. According to previous reports, 12 clinically relevant amino acid positions (118, 120, 126, 129, 130, 133, 134, 141, 142, 143, 144, and 145) were analyzed (25, 32). Positions rtL80, rtI169, rtV173, rtL180, rtA181, rtS184, rtA194, rtS202, rtM204, rtN236T, and rtM250V in the polymerase gene were investigated in order to evaluate treatment resistance mutants for the most widely used antivirals. Additionally, positions 1753, 1762, 1764, and 1896 in the BCP/pC region were studied. Mutations at these positions have been reported to modulate HBeAg expression.

### 2.7. Nucleotide sequence accession numbers

Nucleotide sequences obtained in this work were deposited at the GenBank database under accession numbers: HBV: OM333932 to OM334148 for the S gene and OM456810 to OM456985 for the BCP-pC genomic region; HDV: ON751779.

### 2.8. Statistical analysis

Statistical analyses were conducted using StataMP 14 program. The sociodemographic categorical variables that characterized the sample under study were expressed in proportions stratified by sex. Quantitative values with normal distribution are presented with mean and standard deviation. Data that do not have a normal distribution are presented with median and interquartile ranges. To identify differences between populations, a difference in proportions, a difference in means (t-student), or a difference in medians (W-Mann Whitney) was used according to the distribution of the data, which was determined by means of the normality test (Shapiro–Francia). A level of significance equal to 0.05 was adopted. The strength of the relationship was estimated by using Odds Ratio (CI 95%).

### 2.9. Ethical aspects

This work was evaluated and approved by the Institutional Ethics Committee for Child and Adult Health Research of the Ministry of Health of Córdoba province, Argentina (RePIS N° 2701).

## 3. Results

### 3.1. Clinical and epidemiological characteristics of the studied population

The median age of the population analyzed was 41 years (range 18–73 years) and the 70.5% (153/217) were male. Seventy-nine cases were classified as AHB [(36.4%), mean age 41.7 (± 11.4) years, 67.1% male] and 138 as CHB [(63.6%), mean age 41.3 (± 11.5) years, 66.7% male]. Among AHB individuals, 11 were also human immunodeficiency virus (HIV) (+) [(13.9%), mean age 36.4 (± 9.8) years, 63.6% male] and among CHB, 56 were HBV/HIV co-infected [(40.6%), mean age 38.1 (± 9.7) years, 94.6% male] (Table 1).

**TABLE 1:** Age, gender and hepatitis B virus (HBV) viral load distribution among different stages of HBV infection in mono and HBV/HIV co-infected patients.

Acute hepatitis B (AHB) *N* = 79						
	**HBV mono-infected patients**			**HBV/HIV Co-infected patients**		
		**HBeAg (+)**	**HBeAg (−)/anti-HBeAg (+)**		**HBeAg (+)**	**HBeAg (−)/anti-HBeAg (+)**
N	68	54^c^	14	11	10	1
Age (Mean ± SD)	42.5 ± 11.4	41.2 ± 11.1	47.0 ± 12.5	36.4 ± 9.8	37.1 ± 10.0	29.0
M:F* (M/F Ratio)	46:22 (2.1)	36:18 (2.0)	10:4 (2.5)	7:4 (1.8)	7:3 (2.3)	0:1
**Viral load (UI/ml)**						
Median	3.80E + 05^e^	2.41E + 06	5.48E + 03	3.80E + 07	7.44E + 07	—
**Chronic hepatitis B (CHB) *N* = 138**						
	**HBV mono-infected patients**			**HBV/HIV Co-infected patients**		
		**HBeAg (+)**	**HBeAg (−)/anti-HBeAg (+)**		**HBeAg (+)**	**HBeAg (−)/anti-HBeAg (+)**
N	82	20^c,d^	62	56	48^d^	8
Age (Mean ± SD)	43.5 ± 12.1^a^	43.3 ± 12.3	43.5 ± 12.1	38.1 ± 9.7^a^	37.4 ± 9.4	42.4 ± 10.1
M:F (M/F Ratio)	47:35 (1.3)^b^	16:4 (4.0)	31:31 (1.0)	53:3 (17.7)^b^	45:3 (15.0)	8:0
**Viral load (UI/ml)**						
Median	1.49E + 03^e,f^	1.07E + 08	7.25E + 02	3.34E + 07^f^	5.22E + 07	2.55E + 05

*M, male; F, female.

^a^Age: Chronic hepatitis B (CHB) mono-infection vs. CHB HBV/HIV co-infection *p* < 0.05.

^b^M/F ratio: CHB mono-infection vs. CHB HBV/HIV co-infection *p* < 0.001.

^c^HBeAg (+): Acute hepatitis B (AHB) mono-infection vs. CHB mono-infection *p* < 0.001.

^d^HBeAg (+): CHB mono-infection vs. CHB HBV/HIV co-infection *p* < 0.001.

^e^Median viral load: AHB mono-infection vs. CHB mono-infection *p* < 0.001.

^f^Median viral load: CHB mono-infection vs. CHB HBV/HIV co-infection *p* < 0.001.

Overall, among patients with chronic infection 45.6% (63/138) received antiviral treatment. Among mono-infected patients 30.5% (25/82) were under treatment [9 with entecavir, 9 with tenofovir, 7 changed their treatment regimen to tenofovir after receiving interferon (*n* = 2), lamivudine/3TC (*n* = 2) and entecavir (*n* = 3)], 45.1% (37/82) without treatment and in 24.4% (20/82) data were not available. In HBV/HIV co-infected patients, 67.8% (38/56) were on antiviral treatment regimen with tenofovir, 14.3% (8/56) did not receive therapy, and in 17.9% (10/56) data were not recorded. CHB HBV/HIV co-infected patients had a higher percentage of treatment than those monoinfected with HBV (*p* < 0.05).

In the acute stage, no significant differences in age between HBV-monoinfected patients and HBV/HIV co-infected patients were found (*p* = 0.09). Chronic HBV/HIV co-infected patients were significantly younger than CHB-monoinfected patients (*p* < 0.05). The male to female ratio showed a significant difference between CHB-monoinfected patients (1.3) and CHB-HBV/HIV co-infected patients (17.7) (*p* < 0.001).

The HBeAg positivity rate and the median HBV viral load were significatively higher in: a- AHB vs. CHB among mono-infected patients (3,80E + 05 vs. 1.49E + 03–*p* < 0.001), and b- CHB HBV/HIV co-infected patients vs. CHB mono-infected patients (3,34E + 07 vs. 1.49E + 03–*p* < 0.001) (Table 1).

In all cases, HBV viral loads were significantly higher in HBeAg-positive patients (Table 1).

### 3.2. Genotype and sub-genotype distribution

**FIGURE 1:**
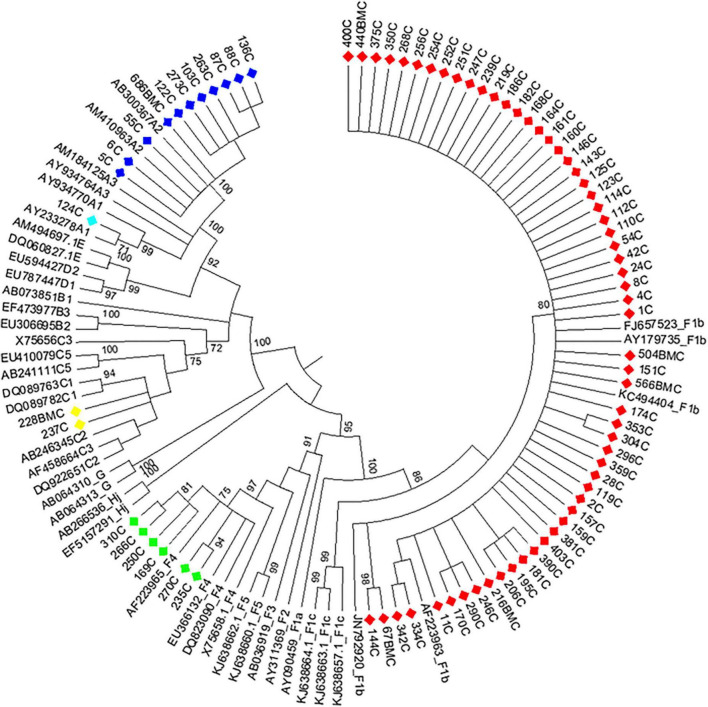
Maximum likelihood phylogeny of acute hepatitis B samples from Córdoba, Argentina. Maximum likelihood phylogenetic tree obtained using sequences of 1,169 nucleotides, resulting from concatenated sequences of the S (471 nucleotides) and BCP-pC genomic regions (698 nucleotides) of acute hepatitis B samples from central Argentina and reference sequences from each genotype or subgenotype available at GenBank. The phylogenetic tree was constructed with MEGA v.6 (bootstrap: 1000 replicates). References: A1, light blue; A2, blue; C, yellow; F1b, red; F4, light green.

**FIGURE 2:**
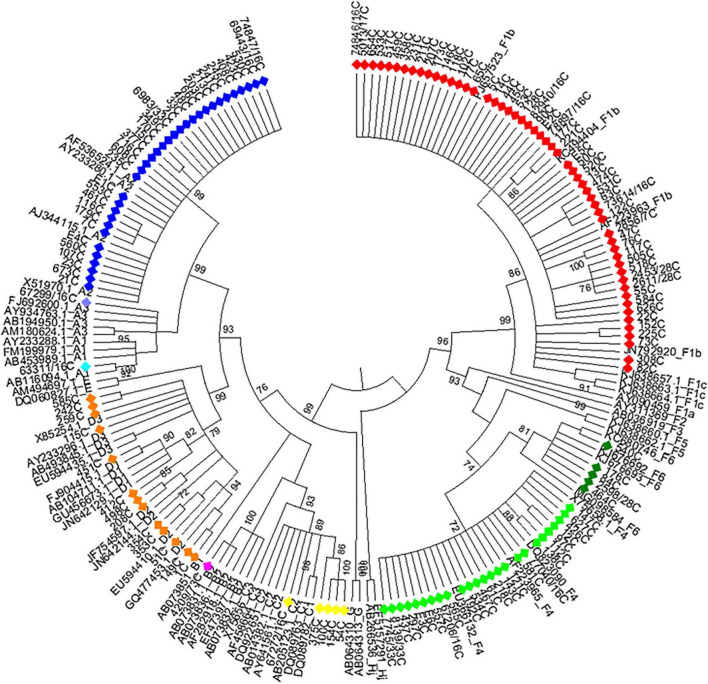
Maximum likelihood phylogeny of chronic hepatitis B samples from Córdoba, Argentina. Maximum likelihood phylogenetic tree obtained using sequences of 1,169 nucleotides, resulting from concatenated sequences of the S (471 nucleotides) and BCP-pC genomic regions (698 nucleotides) of chronic hepatitis B samples from central Argentina and reference sequences from each genotype or subgenotype available at GenBank. The phylogenetic tree was constructed with MEGA v.6 (bootstrap: 1000 replicates). References: A1, light blue; A2, blue; A3, lavender; B, fuchsia; C, yellow; D, orange; F1b, red; F4, light green; F6, green.

Phylogenetic analysis of the S and BCP/pC genomic regions allowed genotyping 100.0% of the samples and subtyping 97.2% (Figures 1, 2). The subtype could not be defined in all samples belonging to gt C (Figures 1, 2) for this reason; this genotype was considered as a whole in subsequent analysis). For gt D, 2 samples grouped within sgt D1, 7 within sgt D2 and in 4 samples the viral subtype could not be defined (Figure 2). Due to the low number of samples of each genotype D subtype, it was also considered as a whole in subsequent analysis.

The overall genotypes/sub-genotypes distribution for the study cohort was as follows: A1 (0.9%), A2 (20.3%), A3 (0.45%), B2 (0.45%), C (3.2%), D (6.0%), F1b (52.1%), F4 (14.3%) and F6 (2.3%).

### 3.3. Genotype distribution in AHB patients

**FIGURE 3:**
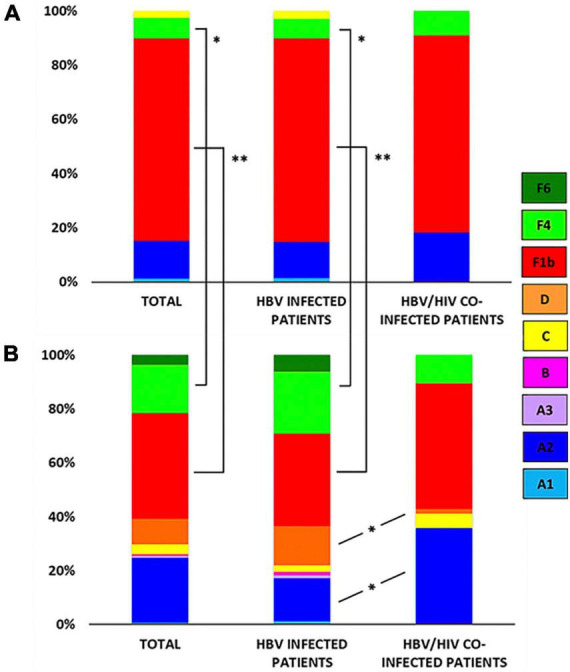
Genotype/sub-genotype distribution in acute hepatitis B (AHB) and chronic hepatitis B (CHB) patients. **(A)** Genotype/sub-genotype distribution in AHB patients (left), AHB monoinfected patients (center), and acute HBV/HIV co-infected patients (right). **(B)** Genotype/sub-genotype distribution in CHB patients (left), CHB monoinfected patients (center), and chronic HBV/HIV co-infected patients (right). References: A1, light blue; A2, blue; A3, lavender; B, fuchsia; C: yellow; D: orange; F1b: red; F4: light green; F6: green; *p < 0.05; ^**^p < 0.001.

Among AHB patients, we found the following genotypes: F (82.3%) > A (15.2%) > C (2.5%). In the same way, HBV sub-genotypes were in the following proportions: F1b (74.7%) > A2 (13.9%) > F4 (7.6%) > C (2.5%) > A1 (1.3%) (Figure 1). No significant differences were observed in gt/sgt distribution between HBV-mono-infected and HBV/HIV co-infected patients in the acute stage (Figure 3A and Supplementary Table 2).

No significant differences were observed among HBV viral loads among patients infected with different HBV gts, in both HBV mono-infected patients and HBV/HIV co-infected patients (*p* > 0.05) (Supplementary Table 2).

### 3.4. Genotype distribution in CHB patients

Among CHB patients the HBV genotype proportion was: F (60.9%) > A (25.4%) > D (9.4%) > C (3.6%) > B (0.7%). In the same way, HBV sub-genotypes were in the following proportions: F1b (39.1%) > A2 (24%) > F4 (18.2%) > D (9.4%) > C and F6 (3.6% each) > A1, A3 and B2 (0.7%) (Figure 2). Sub-genotypes distribution was different between mono- and co-infected patients (Figure 3B). In the 82 HBV-mono-infected sgt F1b was present in 34.1% of cases, followed by sgt F4 (23.2%) and A2 (15.9%). The gt D, sgt F6 and gt C were present in 14.6, 6.1, and 2.4% of cases, respectively. Sgt A1, A3, and B2 were observed in 1.2% of cases (Figure 3B and Supplementary Table 1). Among the 56 HBV/HIV co-infected patients, samples grouped as follows: F1b (46.4%), A2 (35.7%), F4 (10.7%), C (5.4%), and D (1.8%) (Figure 3B and Supplementary Table 2). Among the most prevalent gt/sgt, a significant difference was only found in the distribution between HBV-mono-infected and HBV/HIV-co-infected for sgt A2 (15.9 vs. 35.7%, *p* < 0.05) and for gt D (14.6% vs. 1.8%, *p* < 0.05) (Figure 3B and Supplementary Table 2).

No significant differences were observed among HBV viral loads among patients infected with different HBV gts, in both, HBV mono-infected patients as well as HBV/HIV co-infected patients (*p* > 0.05) (Supplementary Table 2). However, statistically significant differences were only observed in mono-infected CHB patients between genotypes A and D (7.37E + 03 vs. 5.11E + 02–*p* < 0.05).

### 3.5. Comparison between genotype distribution among AHB and CHB patients

Although sgt A2 was more prevalent among CHB patients compared to AHB patients, no significant differences between the two groups were found (Figures 3A, B and Supplementary Table 1). The distribution of sub-genotypes F was significantly different exclusively between AHB and CHB mono-infected patients. A higher detection frequency of F1b (74.7%) was found in AHB than in CHB (39.1%) (*p* < 0.001), and lower F4 (7.6%) frequency in AHB than in CHB (18.1%) (*p* < 0.05). Sgt F6 and gt D were only described in CHB patients (Figures 3A, B and Supplementary Table 2).

### 3.6. Liver injury and genotype distribution among CHB

In 30 patients with CHB infection and available liver tissue [66.7% (20/30) male, mean age 41.8 (± 12.0) years, 20.0% (6/30) HBV/HIV co-infected], the degree of fibrosis (F0-4) was recorded. Nine of them registered grade > F2 (median HBV viral load = 7.9 log10 UI/mL), seven had grade F1 (median HBV viral load = 4.1 log10 IU/mL), and the remaining patients registered stage F0 (median HBV viral load = 3.1 Log10 IU/ml). Five patients presented elevated transaminase levels, all with liver fibrosis stages F3-F4 (Supplementary Table 3).

The general distribution of genotypes among these patients was: F (53.3%) > A (30.0%) > D (13.3%) > C (3.3%). In the group of subjects with liver fibrosis > F2, the genotypes found were: A (55.6%) > F (44.4%), while among the patients with liver fibrosis F0 and F1, genotypes were: F (57.2%) > D (19.0%) = A (19.0%) > C (4.8%).

A significant association was found between the degree of recorded liver fibrosis and inflammatory activity in liver tissue (*p* < 0.001), elevated liver enzymes (*p* < 0.001), advanced age (*p* < 0.05), and HBV viral load (*p* < 0.05). However, no significant association was found between liver fibrosis and gender (*p* = 0.56), viral genotype (*p* = 0.20), treatment (*p* = 0.20) or serological status against HIV (*p* = 0.68) (Supplementary Table 3).

### 3.7. Detection of mutations

#### 3.7.1. Mutations modulating HBeAg expression

The frequency of mutations in the ORF-pC/C region found was 35.5% (77/217): in AHB patients, the frequency was 20.3% (16/79), while in CHB individuals, it was 44.2% (61/138).

Table 2 shows the frequency of mutations in this genomic region and the infecting genotype in the studied groups.

**TABLE 2:** Frequency of mutations in the open reading frame (ORF)-pC/C region in patients with acute and chronic hepatitis B virus (HBV) mono and co-infection according to the infecting genotype.

	Acute hepatitis B (AHB)				Chronic hepatitis B (CHB)							
	**HBV**			**HBV/HIV**	**HBV**					**HBV/HIV**		
Genotype/Sub-genotype	C (*n* = 2)	F1b (*n* = 51)	F4 (*n* = 5)	F1b (*n* = 8)	A2 (*n* = 13)	C (*n* = 2)	D (*n* = 12)	F1b (*n* = 28)	F4 (*n* = 19)	A2 (*n* = 20)	F1b (*n* = 26)	F4 (*n* = 6)
T1753C *n* (%)	1 (50.0)	—	2 (40.0)	—	1 (7.7)	—	3 (25.0)	2 (11.1)	2 (10.5)	1 (5.0)^a^	1 (3.8)^b^	3 (50.0)^a,b^
A1762T/G1764A *n* (%)	1 (50.0)	5 (9.8)	—	2 (25.0)	5 (38.5)	2 (100.0)	2 (16.7)	9 (32.1)	3 (15.8)	2 (10.0)	3 (11.5)	—
G1896A *n* (%)	—	5 (9.8)	—	1 (12.5)	—	1 (50.0)	11 (91.7)^c^	7 (25.0)^c,d^	15 (78.9)^d^	—	—	1 (16.7)

*^a^*A2 vs. F4 *p* < 0.05.

*^b^*F1b vs. F4 *p* < 0.001.

*^c^*D vs. F1b *p* < 0.05.

*^d^*F4 vs. F1b *p* < 0.05.

**FIGURE 4:**
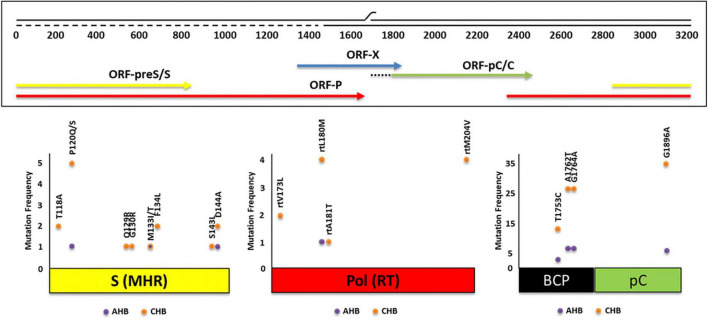
Absolute frequency of mutations obtained for three genomic regions of hepatitis B virus (HBV) [open reading frame (ORF)-S, ORF-P, ORF-pC/C] of 217 patients with acute (AHB) and chronic (CHB) infection. Some of the mutations were found simultaneously.

Within AHB patients, 10.1% (8/79) had the double core mutation A1762T/G1764A, 3.8% (3/79) presented the core mutation T1753C and 7.6% (6/79) had the precore mutation G1896A, mainly observed in HBV-mono-infected patients (Figure 4). These mutations were only found in gts F and C (Table 2). The double mutation A1762T/G1764A and the mutation G1896A were mainly present in sgt F1b, in both HBV-mono-infected and HBV/HIV co-infected AHB patients (Table 2). Besides, the 50% of the individuals with AHB with these mutations were negative for HBeAg with presence of anti-HBe.

For the group of CHB patients, 25.4% (35/138) had the precore G1896A mutation, 18.8% (26/138) had the double core mutation, and 9.4% (13/138) presented the core mutation T1753C. Eight subjects had the double mutation A1762T/G1764A and the precore G1896A mutation simultaneously (Figure 4). As in AHB patients, mutations were mainly present in HBV-mono-infected patients (Table 2). In CHB mono-infected patients, the mutation T1753C was more prevalent in gt D (25.0%) (Table 2), although no significant differences were observed compared to the other gts. The mutation G1896A was found in a significantly higher proportion in gtD and sgt F4 (91.7 and 78.9%, respectively) compared to sgt F1b (25.0%) (Table 2). In HBV/HIV co-infected patients, the frequency for the mutation T1753C reached 50% for sgt F4, significantly higher than the frequency obtained for sgts A2 (5%) and F1b (3.8%) (Table 2). In this group of patients, the mutation G1896A was found only in one subject infected with sgt F4. For all the CHB individuals, the double mutation A1762T/G1764A was found in more than the 10% of the patients, and this frequency was even higher for gt C (40.0%–2/5), sgt F1b (22.2%–12/54), sgt A2 (21.2%–7/33), gt D (16.7%–2/12) and sgt F4 (12.0–3/25%) (Table 2). All samples with G1896A mutation and the 85% of the samples with the double mutation A1762T/G1764A were HBeAg (–), with presence of anti-HBe, respectively.

#### 3.7.2. Mutations related to vaccine escape and diagnostic failure

Overall, ORF-S mutations were detected in the 7.8% of the samples (17/217). Single mutations were observed in 16 cases and double mutations in 1 case. Mutants associated with diagnostic failure and vaccine escape were detected in both, AHB (3.8%) and CHB (10.1%) patients (Figure 4). HBV gt D showed the highest mutation frequency (38.5%), followed by gts C (14.3%), A (8.5%), and F (4.7%) [(gt D vs. gt A, *p* < 0.05) (gt D vs. gt F, *p* < 0.001)]. Mutations were observed in 8 out of 12 aa residues analyzed. The most common mutated residues were P120Q/S (2.8%), D144A (1.4%), T118A, M133T/I, F134L (0.9% each), and Q129R, G130R, and S143L (0.5% each), while 4 positions (I/T126, K141, P142, and G145) did not change. Among the most frequent mutations, P120Q/S mutation was mainly present in gt F, T118A and D144A were exclusively present in gt D and sgt A2, respectively (Table 3).

**TABLE 3:** Frequency of mutations in the open reading frame (ORF)-S region in patients with acute and chronic hepatitis B virus (HBV) mono and co-infection according to the infecting genotype.

	Acute hepatitis B (AHB)		Chronic hepatitis B (CHB)							
	**HBV**	**HBV/HIV**	**HBV**					**HBV/HIV**		
Genotype/Sub-genotype	F1b (*n* = 51)	A2 (*n* = 2)	A2 (*n* = 13)	C (*n* = 2)	D (*n* = 12)	F4 (*n* = 19)	F6 (*n* = 5)	A2 (*n* = 20)	D (*n* = 1)	F4 (*n* = 6)
T118A *n* (%)	—	—	—	—	2 (16.7)	—	—	—	—	—
P120Q/S *n* (%)	1 (2.0)	—	—	—	—	3 (15.8)	—	—	1 (100.0)	1 (16.7)
Q129R *n* (%)	—	—	—	—	1 (8.3)	—	—	—	—	—
G130R *n* (%)	—	—	—	—	—	1 (5.3)	—	—	—	—
M133I/T *n* (%)	—	1 (50.0)	—	1 (50.0)	—	—	—	—	—	—
F134L *n* (%)	—	—	—	1 (50.0)	—	—	1 (20.0)	—	—	—
S143L *n* (%)	—	—	—	—	—	—	—	—	1 (100.0)	—
D144A *n* (%)	—	1 (50.0)	1 (7.7)	—	—	—	—	1 (5.0)	—	—

#### 3.7.3. Antiviral drug-resistance mutations analysis

Amino acid substitutions associated with resistance to antiviral therapy were detected in ORF-P in low frequency (3.2%, 7/217). The most common mutated residues were L180M (2.3%, 5/217), M204V (1.8%, 4/217), V173L (0.9%, 2/217), and A181T (0.5%, 1/217), while seven positions (rtL80, rtI169, rtS184, rtA194, rtS202, rtN236T and rtM250V) did not change (Figure 4). The rtL180M mutation, a possible cause of resistance to LMV and LdT, was detected in one AHB patient (Figure 4). In CHB patients with HBV/HIV co-infection, the following combinations of mutations were detected: rtV173L + rtL180M + rtM204V (reported to cause resistance to LMV, LdT and ETV) and rtL180M + rtM204V (reported to cause resistance to LMV and LdT) and the mutation rtA181T, a cause of resistance to ADV. The mutation V173L was registered in one CHB HBV mono-infected patient (Figure 4). Mutations related to antiviral drug-resistance were only found in patients infected with sgts A2 and F4.

### 3.8. Hepatitis D virus

**FIGURE 5:**
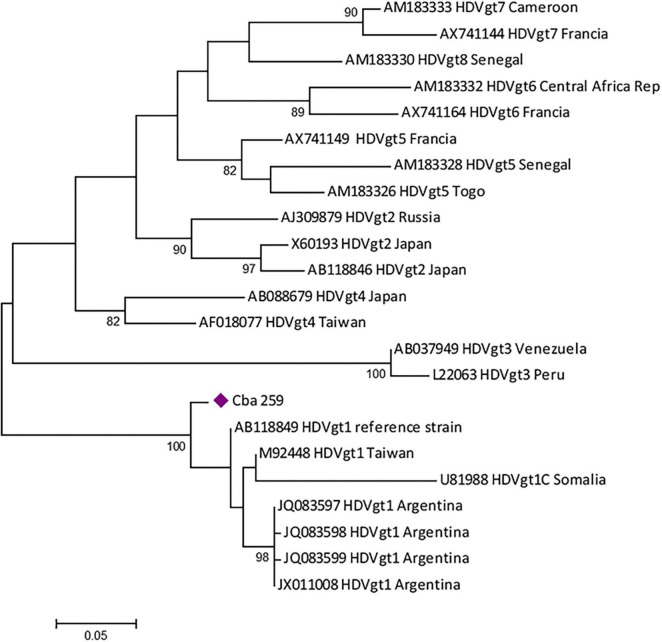
Maximum likelihood phylogenetic tree obtained using sequences of 326 nucleotides, corresponding to the partial HDAg region of hepatitis D virus (HDV), constructed with MEGA v.6 (bootstrap: 1000 replicates). The tree includes the positive sample obtained (violet diamond) and the reference sequences from each genotype available at GenBank.

Four patients were reactive for anti-HDV antibody detection (5.2%, 4/77) and HDV RNA was detected in one sample from a CHB patient with reactive serology. The phylogenetic analysis showed that the sequence obtained belonged to HDV gt 1 (Figure 5), in co-infection with HBV gt D. In the remaining 3 samples IgG anti-HDV + /HDV RNA-, (1 patient with AHB and 2 with CHB), the infecting HBV genotype was F.

## 4. Discussion

In Latin America molecular epidemiology and genomic studies of HBV are restricted to some countries. HBV genotypes A, D, and F are the most frequently detected in South America, but other genotypes have also been observed (B, C, E, G, and H) (33, 34).

The first studies performed in Argentina showed high circulation of genotype F, particularly in northern regions, where there is a large number of people of Amerindian ethnic origin. On the other hand, in large cities, such as Buenos Aires, which are cosmopolitan regions, with a large movement of local people and persons from other countries (immigration and tourism), the genotypes described have been varied, finding similar proportions of gts A, F, and D (18). In addition, in places with particular immigration patterns, such as in Misiones province, with high immigration from Eastern Europe, a preponderance of gt D was recorded (20). In recent years, the gt F has been the most frequently reported in all the provinces, mainly represented by sgts F1b and F4, followed by genotype A, mainly sgt A2 (19–26). Besides, a new variant of gt F emerged, identified as a new F sub-genotype, proposed as sgt F6 (27), showing the importance of continuous genomic surveillance and the study of its biological and clinical implications.

The present study provides information about the molecular profile of HBV and the epidemiological characteristics of patients with acute and chronic hepatitis, treated in the public health system of the province of Córdoba, Argentina, over a period of 8 years. We add updated evidence of the HBV circulating genotypes and sub-genotypes. We determined that genotype F was the most prevalent (68.7%), followed by genotype A (21.6%), in all groups studied: patients with AHB and CHB, mono-infected and co-infected HBV/HIV. A greater diversity of genotypes was found in the group of patients with CHB, compared to AHB. These results agree with most of the countries of South America, in which genotype F is the most prevalent found, since it is autochthonous from the continent (33).

The circulation profile of genotype F among acute infections presented values similar to those found historically in the northern provinces of our country and more recently in Buenos Aires (>80%) (26, 35), and higher than what was previously found in Córdoba (∼50%) (19). Similarly, genotype F was the most frequent in patients with CHB, and its proportion also increased in our region (60.9 vs. 46.7% found in a previous study) (19). The high circulation of the F genotype, particularly the sub-genotype F1b, in our region would then be related to its autochthonous origin and would be the consequence of a late reintroduction from areas enriched with the F genotype (northern provinces and neighboring countries) that took place from the late 1940s to the present (35). Our region is influenced by a constant flow of students or people seeking better working conditions from northern Argentina and from neighboring countries such as Bolivia, Paraguay and Peru, which also leads to the introduction of new viral variants (33, 35, 36).

Sub-genotype F4 was mainly detected in CHB. This sub-genotype has been widely detected in Paraguay, in a frequency of 80%, and is indigenous from South America (36).

The recently described sub-genotype F6 is, at the moment, exclusive to Argentina (27), and was found in our study only in CHB mono-infected patients, as previously described (19, 27).

The second genotype most frequently found in our study cohort, both in AHB and CHB patients, was A, represented mostly by sub-genotype A2. This agrees with previous studies in our country (19, 25). This sub-genotype has been associated to HBV/HIV co-infection, as demonstrated in previous reports (19, 37, 38). Accordingly, in this study the frequency of sub-genotype A2 was higher in HBV/HIV co-infected patients than in mono-infected patients. The precise reason why the sub-genotype A2 is more prevalent in HBV/HIV co-infected patients is unclear. This could be due to risky sexual behaviors in closed or semi-closed social groups. Besides, a shift in the profile of genotypes detected in co-infected patients was observed. Previous studies reported genotype A as the most frequently found in Central Argentina in this group, followed by F (19). Currently, this distribution is inverted, finding genotype F in greater proportion, followed by A. This could be due to inherent differences in sampling among the studies (sample size, geographical distribution of samples) and/or due to a global increase in the dissemination of genotype F in our region.

As previously reported in our area, genotypes B, C and D were found in low frequencies. Genotypes B and D were only detected in patients with CHB. Genotypes B and C are usually associated with the immigration of people from Southeast Asia, and genotype D with immigration from Europe and Eastern Europe (39, 40). Although genotype D is the most frequently detected in the Southern region of Brazil (a limiting country with Argentina), this has not yielded a shift in the HBV genotype profile in our region so far. In fact, some of the patients infected with genotypes B, C, and D included in this study were immigrants from Europe and Asia. These genotypes are rarely detected or detected in low frequencies in our region, so it is inferred that they do not have characteristics of high transmissibility among our population, or have evolutionary disadvantages compared to the two preponderant genotypes F and A.

Various studies have shown the existence of dissimilar characteristics between the different viral genotypes, in relation to the clinical course, HBV viral load, and, particularly, to the HBeAg seroconversion rate (41–43). In our study we only found that individuals infected with genotype A presented a significantly higher HBV viral load than those infected with genotype D among CHB patients. No significant differences between the rest of the clinical features analyzed and the genotypes were found. No conclusions can be drawn in this regard due to the large proportion of genotype F and the low number of samples analyzed for other genotypes, added to the scarce record of clinical variables available in this study.

Hepatitis B virus evolution occurs through mutation and recombination processes (44, 45). The existence of mutations with clinical implications in the HBV genome poses a challenge for the design of diagnostic assays and treatment strategies, and is considered as a potential threat to long-term success of vaccination programs (46, 47). The analysis of nucleotide and amino acid substitutions showed that the observed changes occurred in both acutely and chronically infected patients, mono-infected (HBV) and co-infected (HBV/HIV), but with a higher prevalence in chronic mono-infected patients.

In this study, immune escape mutations were detected in 7.8% of the HBV sequences analyzed, similar to those described in other cohorts from Argentina (7.5–10.7%) (25), China (9.0%) (47), and Spain (6.6–12.5%) (48). Unlike what was found in previous analyzes (32, 49), only 2 of the 12 analyzed aa residues had a mutation frequency greater than 1% (P120Q/S, 2.8%; D144A, 1.4%) and the rest were below this value. In addition, similar to that observed in Argentine patients (25), this study found a significantly higher rate of escape mutations in genotype D (30.8%) than in genotype F (4, 7%).

The most frequently detected HBV mutation with proven vaccine escape properties is the G145R mutation (32). Although other putative escape variants included in the analysis, such as P120T/A and D144A, have been reported (32, 50), evidence for the escape role of these variants is incomplete. Despite the continuous detection of these vaccine escape variants in different parts of the world (32, 50, 51), their dissemination in the population and the consequent reduction in the efficacy of anti-HBV vaccination programs have not generated, to date, a problem that threatens public health, even in areas of high prevalence (52, 53). These mutants involved in immune evasion were found in very low frequency among AHB patients in this study, in accordance with the described by Rodrigo et al. (26), in the metropolitan area of Buenos Aires. Mutation G145R and P120T/A variant, as well as mutations involved in diagnostic failure, were not identified among genotype F (predominant in the region) in our cohort of patients, suggesting that mutations in the ORF-S are not a matter of major concern at this moment.

Mutations in the BCP/pC region associated with a higher risk of HCC, higher HBV viral loads and the decrease or absence of HBeAg (which would contribute to HBeAg seroconversion) have been described (18, 54–57). In our samples, mutations in this genomic region were mainly found in CHB patients. Mutation G1896A was found in a higher proportion in genotypes D and F4 among CHB mono-infected patients, associated to HBeAg seroconversion, in accordance with previous studies (18).

The goal of the treatment in patients with CHB is to prevent the progression of liver disease, the development of cirrhosis and HCC (58). Different studies have shown that sustained suppression of viral replication is associated with remission of liver disease (59). Prolonged treatments with nucleos(t)ide analogs induce the appearance of mutant HBV strains resistant to the different types of drugs used (60). In our study, amino acid substitutions related to antiviral resistance were rare in the AHB patients. Only one sample had the rtL180M mutation, associated to lamivudine and telbivudine resistance. This is consistent with previous studies, which reported low prevalence of mutations in naïve patients (25). In the population of patients with chronic infection, combinations of antiviral resistance mutations to lamivudine, telbivudine, adefovir and intermediate resistance to entecavir were found. However, these mutations were observed in low frequencies and only in HBV/HIV co-infected individuals under antiretroviral treatment (HAART regimens that included tenofovir) (one of them had previously been treated with lamivudine). The results are encouraging for two reasons: (1) there is low circulation of strains with antiviral resistance mutations, and (2) most of the drugs for which resistance was found are not currently the first-line treatment of choice. However, the continuous surveillance of these circulating variants is recommended, due to the possible appearance of mutations with resistance other drugs currently used.

This work reports, for the first time, the circulation of HDV in the central region of Argentina. We found a prevalence of total anti-HDV similar to the previously reported worldwide (61). The sequenced sample belonged to HDV-1, in accordance with previous studies performed in Amerindians from Misiones province (35) and in blood donors of Buenos Aires (22). However, association with HBV sub-genotype D3 was not previously reported in our country. Although HDV infection has been associated with a worse clinical outcome, in our cohort, HDV infection was observed in non-hospitalized subjects. The presence of anti-HDV antibodies in one subject with AHB could indicate a co-infection (or simultaneous infection) with HBV and HDV. More studies including a larger number of samples and the follow-up of the patients over time are needed to elucidate the impact of HDV in our area.

Some limitations in our study need to be considered: (1) we did not use next generation sequencing (NGS) techniques for sequencing samples (we only used Sanger method), so it was not possible to detect and study quasispecies; (2) the lack of full-length sequence data; (3) no data about vaccination were collected in this study. Taking into account the mean age of our cohort and that the vaccination programs anti-HBV in Argentina started in 2000, it is very likely that the great majority of included patients were unvaccinated; in this sense, reinforcing vaccination campaigns in adults is a priority; (4) complete clinical (including markers of liver disease) and therapeutic data were not available for all samples studied. Particularly in the case of positive HDV samples, there were no liver histological data or the clinical evolution of the patients; and (5) the potential lack of generalizability to other regions in South America.

In conclusion, we detected a slight increase in the circulation of genotype F our region, particularly sub-genotype F1b. The high frequency of detection of this genotype among AHB patients suggests transmission advantages over the other genotypes. A low rate of mutations was detected, especially those with antiviral resistance implications, which is an encouraging result. The evidence of HDV circulation in our region (reported for the first time) alerts the health system for its search and diagnosis. These results give scientific evidence of the HBV and HDV circulation in South America, providing valuable information that could be used for health effectors, as well as for the design of treatment guidelines (with particular interest in HBV genotype F). This becomes relevant in the framework of the Strategy for the elimination of viral hepatitis proposed by the WHO for 2030 (62), which urges countries to promote knowledge to guide responses, especially in countries where these infections are poorly studied.

## Data availability statement

The data presented in this study are deposited in the GenBank repository (https://www.ncbi.nlm.nih.gov/genbank/), accession numbers OM333932 to OM334148, OM456810 to OM456985, and ON751779.

## Ethics statement

The studies involving human participants were reviewed and approved by Institutional Ethics Committee for Child and Adult Health Research of the Ministry of Health of Córdoba Province, Argentina (RePIS N° 2701). Written informed consent for participation was not required for this study in accordance with the national legislation and the institutional requirements.

## Author contributions

GC, MP, and VR conceived and designed the experiments. GC, MS, PS, MR, and MP performed the experiments. GC, MP, VR, and AC analyzed the data. CM, RC, JD, and MB contributed reagents, materials, and analysis tools. GC, JD, MP, and VR wrote the manuscript. RC carried out the clinical characterization of the patients. All authors contributed to the article and approved the submitted version.

## Footnotes

1
http://hbv.bioinf.mpi-inf.mpg.de/
