# Supplementary material for: Acute and chronic HBV infection in central Argentina: High frequency of sub-genotype F1b, low detection of clinically relevant mutations and first evidence of HDV
Source: Front Med (Lausanne). 2023 Jan 9;9:1057194. doi: 10.3389/fmed.2022.1057194 (PMC9868314; doi:10.3389/fmed.2022.1057194)
Supplement: Supplementary file 1 [file Table_1.DOCX]

Supplementary Material

**Supplementary Table S1:** Sequences of the primers used in the Nested-PCRs used for HBV and HDV amplification.

| Genomic region | *Primer* | Position (nt) | Sense | Sequence (5´🡪 3´) |
| --- | --- | --- | --- | --- |
| HBsAg | HBV 27 | 57-75 | Sense | CTG CTG GTG GCT CCA GTT C |
|  | HBV 26 | 814-794 | Antisense | AGA AAA TTG GTA ACA GMG GYA |
|  | HBV 29 | 205-226 | Sense | GCG GKG TKT TTC TTG TTG ACA A |
|  | HBV 28 | 789-769 | Antisense | GGG ACT CAA GAT GYT GYA CAG |
| BCP/pC | HBV73 | 1608-1627 | Sense | ATG GAG ACC ACC GTG AAC GC |
|  | HBV2 | 2482-2463 | Antisense | CCC ACC TTA TGA GTC CAA GG |
|  | HBV53 | 1639-1660 | Sense | TGC CAA CAG TCT TAC ATA AGM G |
|  | HBV54 | 2381-2359 | Antisense | GAG TTC TTC TTC TAG GGG ACC TG |
| HDAg | HDV +1 | 853-1267 | Sense | CGG ATG CCC AGG TCG GAC C |
|  | HDV -1 | 1267-1244 | Antisense | GAA GGA AGG CCC TSG AGA ACA AGA |
|  | HDV +2 | 889-910 | Sense | CAT GCC GAC CCG AAG AGG AAA G |
|  | HDV -2 | 1241-1218 | Antisense | TCA GCA AGG AGG AAG AAG AGG AAC |

**Supplementary Table S2:** Genotype distribution among AHB and CHB mono and co-infected patients.

|  |  | Genotype | Sub-genotype | n (%) | Age (mean ± SD) | HBeAg (+) n (%) |  | Viral Load (UI/ml)  Median |
| --- | --- | --- | --- | --- | --- | --- | --- | --- |
| AHB | HBV MONO-INFECTED PATIENTS | Total |  | 68 (86.1) | 42.5 ± 11.4 | 54 (79.4) |  | 3.80E+05 |
|  |  | A (n=10) | A1 | 1 (1.5) | 46 ± 0 | 1 (1.8) | A | 3.27E+06 |
|  |  |  | A2 | 9 (13.2) | 43.8 ± 5.8 | 7 (13.0) |  |  |
|  |  | C (n=2) |  | 2 (3.0) | 46.0 ± 7.1 | 1 (1.8) | C | 1.46E+04 |
|  |  | F (n=56) | F1b | 51 (75.0)^c^ | 42.5 ± 12.3 | 40 (74.1) | F | 4.71E+05 |
|  |  |  | F4 | 5 (7.3)^d^ | 34.8 ± 8.5 | 5 (9.3) |  |  |
|  | HBV/HIV CO-INFECTED PATIENTS | Total |  | 11 (13.9) | 36.4 ± 9.8 | 10 (90.9) |  | 3.80E+07 |
|  |  | A (n=2) | A2 | 2 (18.2) | 41.0 ± 8.5 | 2 (20.0) | A | 7.65E+05 |
|  |  | F (n=9) | F1b | 8 (72.7) | 35.5 ± 10.9 | 7 (70.0) | F | 9.22E+07 |
|  |  |  | F4 | 1 (9.1) | 34.0 ± 0.0 | 1 (10.0) |  |  |
| CHB | HBV MONO-INFECTED PATIENTS | Total |  | 82 (59.4) | 43.5 ± 12.1 | 20 (24.4)^e^ |  | 1.49E+03 |
|  |  | A (n=15) | A1 | 1 (1.2) | 46 ± 0 | 1 (5.0) | A | 7.37E+03^f^ |
|  |  |  | A2 | 13 (15.9)^a^ | 44.8 ± 11.8 | 5 (25.0) |  |  |
|  |  |  | A3 | 1 (1.2) | 39.0 ± 0 | ─ |  |  |
|  |  | B (n=1) | B2 | 1 (1.2) | 38.0 ± 0 | ─ | B | 4.19E+04 |
|  |  | C (n=2) |  | 2 (2.4) | 36.5 ± 3.5 | 1 (5.0) | C | 6.63E+07 |
|  |  | D (n=12) |  | 12 (14.6)^b^ | 38.9 ± 13.6 | ─ | D | 5.11E+02^f^ |
|  |  | F (n=52) | F1b | 28 (34.1)^c^ | 43.3 ± 12.1 | 13 (65.0) | F | 1.34E+03 |
|  |  |  | F4 | 19 (23.2)^d^ | 46.9 ± 12.1 | ─ |  |  |
|  |  |  | F6 | 5 (6.1) | 43.3 ± 18.7 | ─ |  |  |
|  | HBV/HIV CO-INFECTED PATIENTS | Total |  | 56 (40.6) | 38.1 ± 9.7 | 48 (85.7)^e^ |  | 3.34E+07 |
|  |  | A (n=20) | A2 | 20 (35.7)^a^ | 39.7 ± 10.0 | 18 (37.5) | A | 1.13E+07 |
|  |  | C (n=3) |  | 3 (5.4) | 44.3 ± 13.9 | 3 (6.2) | C | 1.39E+04 |
|  |  | D (n=1) |  | 1 (1.8)^b^ | 47.0 ± 0.0 | 1 (2.1) | D | 1.47E+05 |
|  |  | F (n=32) | F1b | 26 (46.4) | 35.1 ± 8.9 | 21 (43.8) | F | 4.15E+07 |
|  |  |  | F4 | 6 (10.7) | 37.9 ± 9.8 | 5 (10.4) |  |  |
| ^a^ sgt A2: CHB mono-infection vs CHB HBV/HIV co-infection p<0.05 | | | | ^d^ sgt F4: AHB mono-infection vs CHB mono-infection p<0.05 | | | | |
| ^b^ gt D: CHB mono-infection vs CHB HBV/HIV co-infection p<0.05 | | | | ^e^ HBeAg: CHB mono-infection vs CHB HBV/HIV co-infection p<0,001 | | | | |
| ^c^ sgt F1b: AHB mono-infection vs CHB mono-infection p<0.001 | | | | ^f^ Viral load: gt A CHB mono-infection vs gt D CHB mono-infection p<0.05 | | | | |

**Supplementary Table S3:** Clinical-epidemiological characteristics and liver injury associated parameters recorded in thirty study patients.

| Patient | Age | Sex | Liver  Inflammation | Fibrosis  Stage | ALT/AST  Level | Viral Load  (Log_10_) | Treatment | Genotype | HIV |
| --- | --- | --- | --- | --- | --- | --- | --- | --- | --- |
| **1** | 48 | F | MINIMAL | F0 | NORMAL | 2,5 | NO | D | NR |
| **2** | 62 | M | MILD | F4 | NORMAL | 3,5 | NO | A | NR |
| **3** | 47 | M | MINIMAL | F0 | NORMAL | 7,8 | YES | F | NR |
| **4** | 34 | M | MILD | F2 | NORMAL | 3,8 | YES | F | R |
| **5** | 51 | M | MILD | F2 | NORMAL | 8,0 | YES | A | R |
| **6** | 55 | M | MODERATE | F3 | ELEVATED | 8,0 | NO | A | NR |
| **7** | 37 | M | MINIMAL | F0 | NORMAL | 2,8 | NO | A | NR |
| **8** | 58 | M | SEVERE | F4 | ELEVATED | 7,3 | YES | A | R |
| **9** | 47 | F | MILD | F0 | NORMAL | 2,1 | NO | F | NR |
| **10** | 39 | F | MINIMAL | F0 | NORMAL | 3,0 | NO | F | NR |
| **11** | 57 | F | SEVERE | F4 | ELEVATED | 7,9 | NO | F | NR |
| **12** | 47 | F | MINIMAL | F0 | NORMAL | 2,1 | NO | A | NR |
| **13** | 34 | M | MILD | F0 | NORMAL | 8,0 | NO | F | R |
| **14** | 36 | F | MINIMAL | F0 | NORMAL | 4,5 | NO | F | NR |
| **15** | 29 | M | MINIMAL | F1 | NORMAL | 2,2 | NO | D | NR |
| **16** | 24 | F | MINIMAL | F1 | NORMAL | 3,1 | NO | D | NR |
| **17** | 56 | M | MINIMAL | F3 | ELEVATED | 8,5 | YES | A | NR |
| **18** | 35 | M | MINIMAL | F1 | NORMAL | 3,1 | NO | F | NR |
| **19** | 48 | F | MINIMAL | F1 | NORMAL | 4,1 | NO | F | NR |
| **20** | 42 | M | MINIMAL | F0 | NORMAL | 3,0 | ND | D | NR |
| **21** | 26 | M | MINIMAL | F1 | NORMAL | 8,9 | NO | F | R |
| **22** | 58 | M | SEVERE | F4 | ELEVATED | 8,1 | NO | F | NR |
| **23** | 29 | M | MILD | F0 | NORMAL | 4,1 | NO | F | NR |
| **24** | 57 | F | MILD | F2 | NORMAL | 5,6 | YES | F | NR |
| **25** | 26 | F | MINIMAL | F0 | NORMAL | 3,2 | NO | F | NR |
| **26** | 18 | M | MILD | F0 | NORMAL | 2,8 | NO | F | NR |
| **27** | 27 | M | MINIMAL | F1 | NORMAL | 7,9 | NO | A | NR |
| **28** | 46 | M | MINIMAL | F0 | NORMAL | 8,5 | NO | A | NR |
| **29** | 39 | M | MINIMAL | F0 | NORMAL | 8,1 | YES | C | NR |

**ND: No Data, NR: Non-Reactive, R: Reactive, F0: No Fibrosis (absence of scarring), F1: Minimal/mild fibrosis, F2: Significant/moderate fibrosis, F3: Severe fibrosis, F4: Cirrhosis or advanced scarring.**
